# Multi-Beam Steering for 6G Communications Based on Graphene Metasurfaces

**DOI:** 10.3390/s21144784

**Published:** 2021-07-13

**Authors:** Huifang Ai, Qianlong Kang, Wei Wang, Kai Guo, Zhongyi Guo

**Affiliations:** 1 School of Computer and Information, Hefei University of Technology, Hefei 230009, China; aihuifanghfut@163.com (H.A.); m18955890051@163.com (Q.K.); kai.guo@hfut.edu.cn (K.G.); 2 Department of Mathematics and Physics, Shijiazhuang Tiedao University, Shijiazhuang 050043, China; wangw@stdu.edu.cn

**Keywords:** metasurface, multi-beam steering, directional radiation

## Abstract

As communication technology is entering the 6G era, a great demand for high-performance devices operating in the terahertz (THz) band has emerged. As an important part of 6G technology, indoor communication requires multi-beam steering and tracking to serve multi-users. In this paper, we have designed a graphene metasurface that can realize multi-beam steering for directional radiations. The designed metasurface consists of graphene ribbons, dielectric spacer, and metal substrate. By designing the graphene ribbons and controlling the applied voltage on them, we have obtained single-, double-, and triple-beam steering. In addition, we have also numerically calculated the far-field distributions of the steered multi-beam with a diffraction distance of 2 m. Our design has potential applications in future indoor directional 6G communications.

## 1. Introduction

Future 6G wireless communication systems have already attracted tremendous attention [1,2]. Terahertz (THz) communications are among one of the most attracting 6G technologies because it has high capacity and high security compared with microwave communication [3,4]. However, the strong directionality and narrow beam of terahertz waves may easily cause the problem of local signal coverage holes and leads to signal transmission interruption [5,6]. Therefore, a functional device that realizes intelligent control of terahertz beams is urgently needed in the field of terahertz communications, enabling flexible control of the radiation direction and coverage area of the beam according to the actual communication requirement.

Directional radiation is a key component in terahertz communication systems. There have been several different techniques previously employed to develop multi-beam patterns in different spectra, involving the use of phased array antennas [7,8], graded metamaterials based on transformation optics [9,10], and metasurfaces [11,12,13]. Among these approaches, metasurface, an ultra-thin and compact man-made structure, can achieve artificial modulations of electromagnetic wave parameters, including amplitude, phase, polarization, and other characteristics [14,15,16,17,18,19,20]. By providing abrupt geometric or resonant phase discontinuities, the metasurfaces can achieve control of the beam wavefront [21,22,23,24]. In particular, they can steer reflected or transmitted light to an arbitrary anomalous angle, the angle of reflection or transmission can be calculated by using the generalized Snell’s law [25,26,27,28]. Therefore, they can be used as reflective or transmitting array antennas, which are ideal for free-space optical communications. It has been demonstrated that metasurface could be one of the key technologies for the 6G wireless communication systems, such as massive multiple input multiple output technology and smart radio environments [29,30,31]. Recently, reconfigurable metasurfaces based on PIN diodes have been designed for indoor 6G multi-user communications at the terahertz region by controlling the state of the PIN diode [32,33,34]. However, PIN diodes may have losses in the terahertz band, other materials with relatively low loss have also been introduced into metasurfaces for indoor 6G multi-user communication with the help of electric- and polarization-controlling [35,36,37,38]. Nevertheless, the above designs have not discussed the distribution of the beams after propagating far enough (propagation distance is much greater than the wavelength). Hence, a dynamically tunable multi-beam control device based on metasurface can be designed to improve the coverage performance in multi-terminated terahertz communications by studying the beam propagation.

In this paper, we designed a tunable graphene metasurface for far-field multi-beam steering and directional beam propagation in terahertz communication technology. It was shown that the multi-beam steering could be engineered by choosing graphene ribbons with different widths and controlling applied voltage on them. As proofs of concept, we designed three devices for far-field beam steering: 1. steering three-beams at angles of −42°, 13°, and 42°; 2. steering two-beams at angles of −42°, 42°and −42°, 13°; 3. steering a single-beam to an angle of 42°. By studying the near-field distributions and far-field radiation patterns of the multi-beam steering, we demonstrated that our metasurfaces have excellent performances of anomalous multi-beam steering. Our work has paved the way for the application of directional indoor communication.

## 2. Materials and Methods

**Figure 1:**
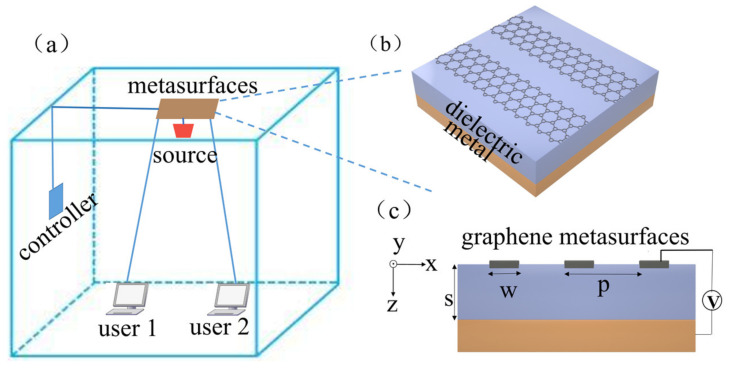
(**a**) Indoor THz communication scheme for multi-user scenarios. (**b**) Schematic of a graphene metasurface that consists of a metal substrate, a dielectric layer, and top graphene ribbons. (**c**) A sectional view of the graphene metasurface. *p* is the grating period, *w* is the width of the graphene ribbon and s is the thickness of the SiO_2_ dielectric layer.

There is a possible application scenario presented here for indoor THz communication. As shown in Figure 1a, the metasurface is stuck on the wall to control the propagation of the reflected waves. By controlling the voltage applied to the metasurface through the controller, the source is converted to multi-beam for multi-user by a metasurface. Figure 1b shows the overview of our designed graphene metasurface. The top layer is graphene ribbons (infinite in the y-axis), whose optical responses are determined by the width in the x-axis and the applied gate voltage. A SiO_2_ layer acts as a dielectric spacer. A metal substrate works as a backside reflector. Figure 1c is a sectional view of the designed graphene metasurface, where *w*, *p*, and s denote the width of graphene ribbons, the structure period, and thickness of the SiO_2_ layer, respectively. The metal substrate is assumed to be thick enough. Via choosing appropriate structural parameters and applied gate voltage, the designed metasurface may sufficiently interact with incident light and allows us artificially steer the wavefront of the THz beam.

To investigate the designed structure, numerical simulations have been performed. In our model, the graphene ribbons are treated as 2D conductors. Its optical properties can be described by the conductivity $\sigma_{g}$, which is calculated by the Kubo model, and the conductivity consists of intra-band conductivity and inter-band conductivity [39]:

(1) $$\sigma_{g}=\sigma_{\mathrm{intra}}+\sigma_{\mathrm{inter}}$$

where *σ*_intra_ and *σ*_inter_ are related to the electron-photon scattering process within the band and the interband transition, respectively. At room temperature T = 300 K, the interband transition can be ignored due to the Pauli exclusion principle, and the conductivity of graphene can be simplified to a Drude-like model [40]:

(2) σ(w)=ie2EFπℏ2(w+iτ)

where *e* is the electron charge, *E_F_* is the Fermi energy level, *ћ* is the Planck constant, *τ* is the relaxation time, which is 10^−12^ s in this work [41,42,43]. When graphene has a thickness, the permittivity of graphene can be simplified as [44]:

(3) $$\varepsilon (w)=1+\frac{i\sigma (w)}{\varepsilon_{0}wt}$$

where *t* is the thickness of the graphene which is 0.35 nm in this work, *ε*_0_ is the permittivity of free space. From the above equations, it is clear that the Fermi energy level of graphene can be easily modulated by the gate voltage [45]. In general, the Fermi levels in graphene can be expressed as $E_{F}=\hbar v_{f}\sqrt{\pi \varepsilon_{SiO_{2}}\varepsilon_{0}V/et}$. Here, the Fermi speed is set as *v_f_* = 10^6^ m/s, and *V* is the applied electric potential. So once we know the Fermi energy level of graphene, we can determine the corresponding voltage according to the formula. Due to the tunability of permittivity of graphene, we can achieve dynamic modulation of the beam deflection by designing a metasurface with a specific phase profile.

**Figure 2:**
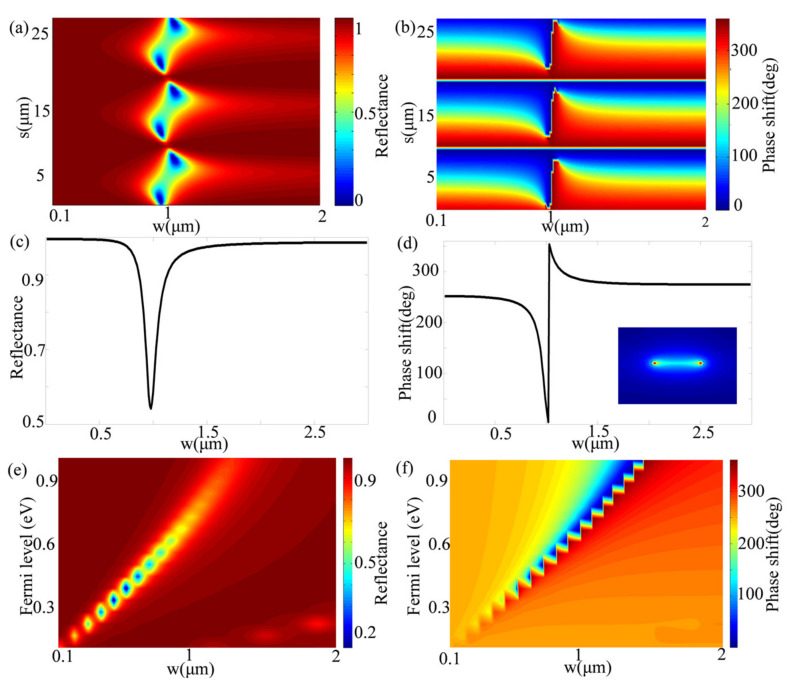
(**a**) Reflectivity and (**b**) phase shift is the function of the graphene ribbon width (*w*) and the dielectric layer thickness (s). (**c**) Reflectivity and (**d**) phase shift vary as the graphene ribbon width (*w*) increasing at a thickness of the dielectric layer (s) as 3 μm. (**e**) Reflectivity and (**f**) phase shift is the function of the graphene ribbon width (*w*) and Fermi energy level (*E_F_*). The inset shows the distribution of the electric field at plasmonic resonance when the graphene ribbon width and the dielectric layers are 1.03 μm and 3 μm, respectively, at the operating frequency of 12.32 THz.

As a proof of concept, we designed a metasurface to operate at 12.32 THz. The operating frequency of the proposed graphene metasurface can be easily reduced by scaling the size of the graphene metasurface as graphene strongly interacts with an electromagnetic wave with low loss in the terahertz regime. We used the finite element method to simulate the performances of the unit cell and the designed metasurfaces. Figure 2a,b shows the reflectivity and reflection phase of the unit cell when the width of the graphene ribbon (*w*) and the thickness of the dielectric layer (s) change, respectively. In these cases, the Fermi energy level of graphene is 0.64 eV. The refractive index of SiO_2_ is 1.4 [46], and the period of graphene (*p*) is 3 μm. The drude model is applied to describe the optical property of a gold-backed reflector with a thickness of 2 μm. The light source is a normal incidence with TM polarization. When the width of the graphene ribbon is near 1 μm, the reflection amplitude varies significantly and phase change almost covers 0–2π, showing the characteristic of plasmon resonance. It can be also observed that the reflectivity and reflection phase will change periodically with the dielectric layer thickness, which may be attributed to Fabry−Perot resonance in the dielectric layer. Herein, we can treat the designed structure as an asymmetric Fabry−Perot resonator, where the graphene ribbons and metal substrate act as front and back reflectors, respectively. According to these results, we can choose a dielectric layer with specific thickness regarding the required reflectivity and reflection phase.

In this work, we chose the SiO_2_ layer thickness of 3 μm. Figure 2c shows that the reflectivity of the designed unit cell reaches more than 55% with the change of the graphene ribbon width. Meanwhile, Figure 2d shows that continuous 2π phase modulation can be achieved by changing the width of the graphene ribbon. The inset in Figure 2d illustrates the electric field distributions of the unit cell at the plasmon resonance. Figure 2e,f show the reflectivity and reflection phase of the unit cell as functions of the width (*w*) and Fermi energy level of the graphene ribbon, respectively. Due to the Fabry–Perot resonance between graphene ribbons and metal substrate, the continuous 2π phase modulation of reflection can be achieved, further demonstrating the desired metasurface can be constructed by simply choosing the values of *w* and *E_F_*.

## 3. Discussion

Based on the results above, we can achieve deflection of the reflected beam at the frequency of 12.32 THz. The designing of the metasurface is mainly based on the generalized Snell’s law, in which phase gradient $\frac{d\varphi }{dx}$ is introduced into the metasurface. The relationship between the reflection angle and the incidence angle can be calculated by the following formula [47]:

(4) $$\sin(\theta_{r})-\sin(\theta_{i})=\frac{\lambda }{2\pi n_{i}}\frac{d\varphi }{dx}$$

where *n*_i_ denotes the refractive index of the medium in the incident region, *θ_i_* is the angle of incidence, *θ_r_* is the angle of reflection. When the incident light is normally incident and the refractive index of the medium in the incident region *n_i_* = 1, the reflection angle can be written as:

(5) $$\theta_{r}=\sin^{-1}\left(\frac{\lambda }{2\pi }\frac{d\varphi }{dx}\right)$$

**Figure 3:**
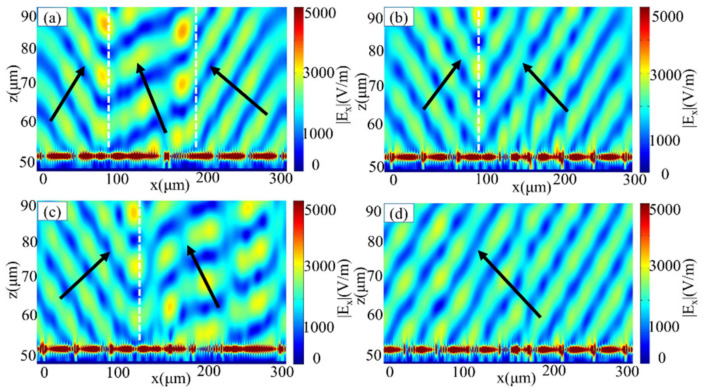
The graphene metasurface was designed to operate at 12.32 THz. (**a**) Reflected electric field Ex-distribution in the three anomalous deflection directions of the metasurface with a Fermi energy level of 0.64 eV. (**b**) Symmetric and (**c**) asymmetric anomalous reflection electric field Ex distribution of two beams, (**d**) anomalous reflection electric field Ex distribution of single beam at fixed structure parameters as various the Fermi energy level of the graphene ribbons.

According to Equation (5), the reflection angle, i.e., the beam steering direction is related to the phase gradient when the incident wavelength is determined. We may achieve multi-beam steering for directional radiations as if we introduced different phase gradients to the metasurfaces. Using this concept, we designed a metasurface to control the normal incident waves and generate multi-beam reflections. We chose 102 graphene ribbons with a period of 3 μm. For the normal incident TM polarized light frequency of 12.32 THz, we first implemented three kinds of phase gradients of −π/6, π/18, and π/6 with 26, 37, and 39 unit cells, respectively. Therefore, the theoretical deflection angles are −42°, 13°, and 42°, which can be calculated from Equation (5). The unit cells are selected appropriately to achieve corresponding phase shifts according to Figure 2c,d. In these cases, the Fermi energy levels of all graphene ribbons are set as 0.64 eV. To demonstrate, we simulated the designed metasurface and the results as shown in Figure 3a. We can easily see the anomalous reflection of three beams from the Ex distributions of the reflection beams. The phase shifts of −π/6, π/18, and π/6 are distributed from left to right. The reflection angles of three-phase shifts are estimated to be −42°, 13°, and 42°, so we can observe three beams with different deflection directions in Figure 3a.

When we fix the structure parameters in Figure 3a, the metasurface can be dynamically modulated by changing the Fermi energy level. Figure 3b–d show that the designed metasurface can achieve beam steering in different directions. From the Drude model of graphene, it is known that the conductivity of graphene is related to the Fermi energy level and can be controlled by the applied voltage. Therefore, when our structure is fixed, desired phase gradient can be achieved by tuning different Fermi energy levels of graphene ribbons. As shown in Figure 3b, we implemented two-phase gradients of −π/6 and π/6 with 39 and 63 unit cells, respectively. Therefore, the incident beam will be deflected to the directions with angles of −42° and 42°, respectively. From the field distribution in Figure 3b, we can clearly observe the deflection in two opposite directions. In addition, we achieve deflection in two asymmetric directions, as shown in Figure 3c. In this case, we choose 39 and 63 graphene ribbons to implemented two kinds of phase gradients of π/6 and π/18, respectively. The theoretical deflection angles are calculated to be −42° and 13°. From the distribution of *E*_x_, we can easily see the anomalous reflection of the two asymmetric beams at the directions with angles of about −42° and 13°. Similarly, by selecting 102 graphene ribbons with a phase gradient of π/6, we achieved a single beam deflection with an angle of about 42°, as shown in Figure 3d. Due to too much data, here we only give the variation of the Fermi energy level at the asymmetric anomalous reflection as shown in Table 1. From Figure 2f, when fixing w, we can achieve the desired phase by choosing a different *E_F_*. From Figure 3a–d, it is clear that by providing the desired phase gradient and then by controlling the Fermi energy level of the graphene ribbons, we can dynamically modulate the metasurface to achieve deflection of multi-beam in different directions, which shows the potential applications for multi-user communications simultaneously.

**Figure 4:**
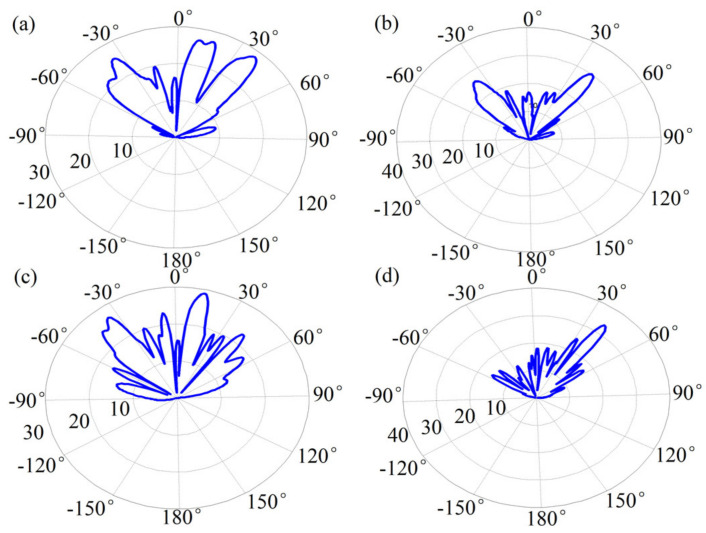
Far-field radiation map of reflected waves. (**a**) Far-field radiation map of three reflected beams. (**b**) Far-field radiation map of two symmetrical reflection beams. (**c**) Far-field radiation map of two asymmetric reflection beams. (**d**) Far-field radiation map of the single reflected beam. The unit is dB.

In the following, we calculated the far-field radiation patterns of our designed metasurfaces. Figure 4a shows the gain of beamforming corresponding to the simulated electric field in Figure 3a. In the far-field region, three peaks with a gain of 25.6 dB, 27.2 dB, and 28.2 dB can be observed at the directions with angles of −37°, 11°, and 39°, which are in good agreement with the theoretical values of −42°, 13°, and 42°, respectively. Note that, the slight deviations between the theoretical and simulated results may be attributed to that the phase shift generated by the graphene ribbons does not completely cover the full 0–2π range. Figure 4b shows the far-field pattern of the results in Figure 3b and the generated beams with a gain of 26.4 dB and 30.1dB are read as −38° and 40°, which are also in good agreement with the theoretical values of −42° and 42°, respectively. Besides the reason in Figure 4a, the deviation also has not been able to accurately match the appropriate Fermi energy level for each graphene band to achieve the corresponding phase gradient. Similarly, Figure 4c shows the beams with a gain of 26.8 dB and 30.1 dB deflected at angles of −35° and 12°, respectively. Figure 4d plots the beam with a gain of 33.5 dB at a deflection angle of 40°. The results in Figure 4 validated that our designed metasurface can work well in the multi-beam deflection for indoor multi-user communication. More importantly, the deflection beams at the far-field region show a narrow beamwidth and avoid signal interferences.

**Figure 5:**
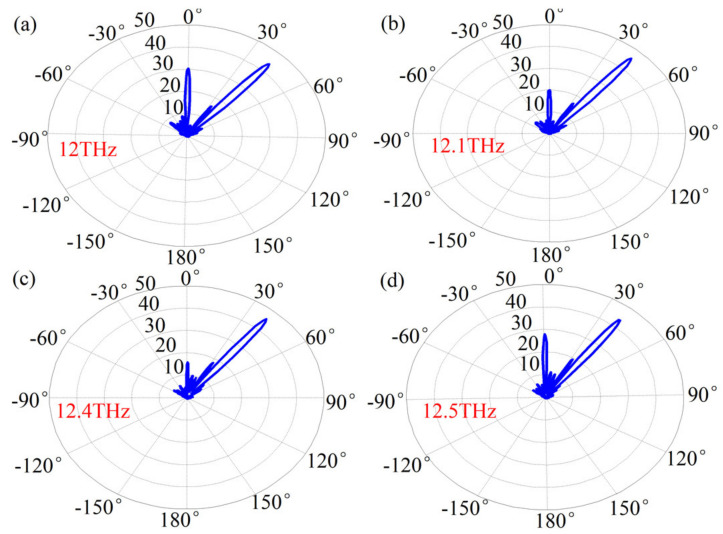
Broadband performances of single-beam directional radiation. The far-field distributions of the electric field at the frequencies of (**a**) 12 THz, (**b**) 12.1 THz, (**c**) 12.4 THz, and (**d**) 12.5 THz. The unit is V/m.

Bandwidth is a key feature to evaluate the performance of communication devices. To demonstrate, we have also simulated beam deflection in the frequency range 12–12.5 THz, and here we give the far-field radiation map for the single-beam case, as shown in Figure 5. When the incident frequency varies from 12 THz to 12.5 THz, a single-beam with high directivity at angles of 40.2°, 41.1°, 41.79°, and 39.4° can be produced through this bandwidth. The deflection angle is not sensitive to the working frequency, validating the good performances of the proposed system. Similar results are obtained for other cases with different beam deflections (not shown).

To demonstrate the performances of our designed metasurface, we applied Fraunhofer’s diffraction formula to simulate the amplitude distribution of multi-beam along the x-direction at a propagation distance of 2 m. The Fraunhofer formula is written as:

(6) $$E(x,y)=\frac{1}{i\lambda z}e^{ik_{0}(z+\frac{x^{2}}{2z})}\iint E(x_{0},y_{0})e^{\frac{-ik_{0}}{z}(x_{0}\cdot x+y_{0}\cdot y)}dx_{0}dy_{0}$$

where *E* is the electric field, (*x*_0_,*y*_0_) are the coordinates on the metasurface plane, *k*_0_ is the wave vector of the incident light in the vacuum, *λ* is the incident wavelength.

**Figure 6:**
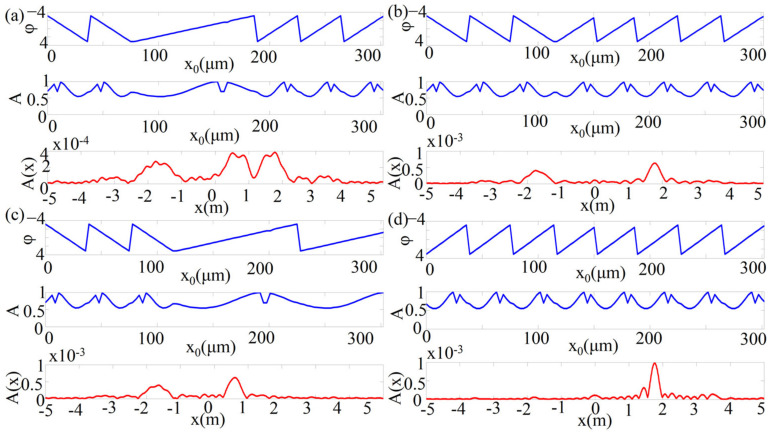
The beam at a propagation distance of 2 m away is calculated by the Fraunhofer diffraction formula. The top and middle parts are the theoretical phase and amplitude of the metasurface. The bottom part is the diffraction amplitude distribution calculated by the Fraunhofer diffraction formula (**a**) Three beams, (**b**) two symmetrical beams, (**c**) two asymmetrical beams, and (**d**) one beam calculated to obtain the distribution of the beams at a propagation distance of 2 m.

In Figure 6a–d, the top and middle panels show the theoretical phase and amplitude distributions on the metasurface plane, respectively. The bottom panels show the diffracted amplitude distributions which are calculated by Equation (6). Figure 6a–d show the amplitude distribution of the electric field obtained by numerical calculation at a propagation distance of 2 m away. As the metasurface length is much smaller than the propagation distance, the metasurface width can be neglected, and then the calculation of the beam angle after 2 m propagation can be simplified. From the bottom panel of Figure 6a, it can be observed that the deflection beams are separated in three different directions. The deflection angles of the three beams can be calculated as −41.98°, 11.3°, and 39.52°. From Figure 6b, it can be observed that the two beams are separated at two different positions, and the deflection angles are calculated to be −41.98° and 40.36°. Similarly, two beams with separation angles of −35° and 12.68° can be obtained in Figure 6c. As the two-phase gradients designed in Figure 6b,c are different, the beams will be separated at different positions. Figure 6d shows the results for only one phase gradient, so a position can be observed and a deflection angle of 41.98° is obtained. Comparing the simple deflection angle calculated in Figure 6 with the deflection angle calculated by Equation (5), it can be seen that the beams deflected by the designed metasurface have good performances after 2 m propagation. The results in Figure 6 further validate the practicality of our metasurface, where multiple beams can be separated at different locations to enable multi-user communication.

## 4. Conclusions

In summary, we introduced a dynamically tunable metasurface based on graphene ribbons for multi-user indoor communication. By properly choosing the width of the graphene ribbons, the continuous 0–2π phase coverage can be achieved. Similarly, the desired phase shift can be obtained by adjusting Fermi energy levels applied to the graphene ribbons. Simulation shows that the designed metasurface can achieve dynamic adjustable multi-beam far-field anomalous reflection. From the near field distributions and the far-field radiation patterns, we can clearly observe the deflection of three beams, two beams, and a single beam. We also numerically calculated the field distribution of multi-beams at a distance of 2 m away, and the results demonstrated that the beams are separated into beams with different propagation angles. Overall, we have designed a metasurface that provides a new method to achieve indoor directional communication.

## Figures and Tables

**Table 1:** The variation of the Fermi energy level for the asymmetric anomalous reflection.

*w*(μm) (1–11)	1.030	1.062	1.157	1.027	0.690	0.862	0.912	0.939	0.957	0.971	0.985
*E_F_*(eV)	0.640	0.640	0.640	0.640	0.640	0.640	0.640	0.640	0.640	0.640	0.640
*w*(μm) (12–22)	1.001	1.020	1.030	1.062	1.157	1.027	0.690	0.862	0.912	0.939	0.957
*E_F_*(eV)	0.640	0.640	0.640	0.640	0.640	0.640	0.640	0.640	0.640	0.640	0.640
*w*(μm) (23–33)	0.972	0.986	1.002	1.020	1.020	1.016	1.008	1.001	0.996	0.991	0.986
*E_F_*(eV)	0.640	0.640	0.640	0.640	0.600	0.597	0.563	0.450	0.617	0.800	0.717
*w*(μm) (34–44)	0.981	0.976	0.972	0.967	0.962	0.957	0.952	0.946	0.940	0.932	0.923
*E_F_*(eV)	0.685	0.656	0.650	0.640	0.620	0.600	0.600	0.598	0.596	0.593	0.590
*w*(μm) (45–55)	0.913	0.900	0.884	0.862	0.832	0.783	0.690	0.390	1.027	1.028	1.477
*E_F_*(eV)	0.586	0.583	0.580	0.556	0.551	0.502	0.450	0.250	0.707	0.716	0.980
*w*(μm) (56–66)	1.242	1.158	1.112	1.083	1.062	1.046	1.034	1.030	1.020	1.002	0.986
*E_F_*(eV)	0.977	0.891	0.856	0.846	0.852	0.880	0.947	0.614	0.613	0.614	0.324
*w*(μm) (67–77)	0.972	0.957	0.940	0.913	0.862	0.690	1.028	1.158	1.062	1.030	1.020
*E_F_*(eV)	0.442	0.490	0.510	0.514	0.500	0.400	0.600	0.700	0.650	0.600	0.650
*w*(μm) (78–88)	1.002	0.986	0.972	0.957	0.940	0.913	0.862	0.690	1.028	1.158	1.062
*E_F_*(eV)	0.650	0.651	0.651	0.610	0.602	0.601	0.553	0.450	0.685	0.805	0.729
*w*(μm) (89–99)	1.030	1.020	1.002	0.986	0.972	0.957	0.940	0.913	0.862	0.690	1.028
*E_F_*(eV)	0.704	0.700	0.693	0.688	0.684	0.677	0.672	0.654	0.625	0.480	0.935
*w*(μm) (100–102)	1.158	1.062	1.030								
*E_F_*(eV)	0.717	0.667	0.610								
